# Primary bladder diffuse large B-cell lymphoma: a rare case report and literature review

**DOI:** 10.3389/fmed.2025.1613673

**Published:** 2025-07-21

**Authors:** Ya Li, Xianwen Hu, Jiong Cai

**Affiliations:** Department of Nuclear Medicine, Affiliated Hospital of Zunyi Medical University, Zunyi, China

**Keywords:** primary bladder lymphoma, Extranodal lymphoma, diffuse large B-cell lymphoma (DLBCL), PET-CT, diuretic

## Abstract

Primary bladder diffuse large B-cell lymphoma (PB-DLBCL) is an exceedingly rare form of non-Hodgkin lymphoma, accounting for only 0.2% of all extranodal lymphomas. Here we report a case of PB-DLBCL in a woman presenting with hematuria. To determine the disease stage, an ^18^F-FDG PET/CT scan was performed after surgery. However, intense physiological ^18^F-FDG activity within the bladder lumen obscured potential lesions and concurrent CT imaging revealed suboptimal bladder distension limiting anatomical delineation. To address these limitations, 20 mg of intravenous furosemide was administered. Delayed imaging was performed 2 h after ^18^F-FDG injection (1 h after diuretic administration), revealing a focal hypermetabolic lesion in the left bladder wall. This metabolic pattern confirmed the diagnosis of PB-DLBCL, staged as IE according to the Ann Arbor classification. Following 4 cycles of R-CHOP chemotherapy, an ^18^F-FDG PET/CT scan was performed for treatment response assessment, confirming complete metabolic remission of the PB-DLBCL. This case highlights that dual-phase ^18^F-FDG PET/CT with intravenous diuretics can reduce bladder background activity and improve lesion visualization. Moreover, this approach holds significant clinical value in assisting with disease staging and evaluating treatment response.

## Introduction

As an exceptionally rare extranodal malignancy, primary bladder lymphoma comprises just 0.2% of all non-Hodgkin lymphoma cases originating outside lymph nodes (1). Extranodal lymphoma diagnosis relies on comprehensive histopathological examination and immunohistochemistry (2). ^18^F-FDG PET/CT combining functional and anatomical imaging, forms the cornerstone of lymphoma patient management (3). PET/CT demonstrates superior accuracy and sensitivity to CT in identifying involved nodal sites (4). However, diagnostic challenges persist in regions with physiological ^18^F-FDG uptake or excretion, such as the brain and urinary tract. We present a rare case of primary bladder DLBCL. Initial ^18^F-FDG PET/CT imaging at 45 min post-intravenous tracer administration revealed that physiological tracer excretion limited lesion visualization. Accurate identification of bladder lesion sites presented significant challenges. To address this limitation, intravenous diuretic administration was implemented to mitigate physiological uptake within the bladder. Following intravenous diuretic administration, delayed imaging performed at 2-h post-radiotracer injection clearly delineated the bladder wall lesion. Compared with post-chemotherapy PET/CT images without diuretics, the diuretic-assisted protocol showed reduced urinary activity and clearer lesion visualization.

## Case presentation

**Figure 1:**
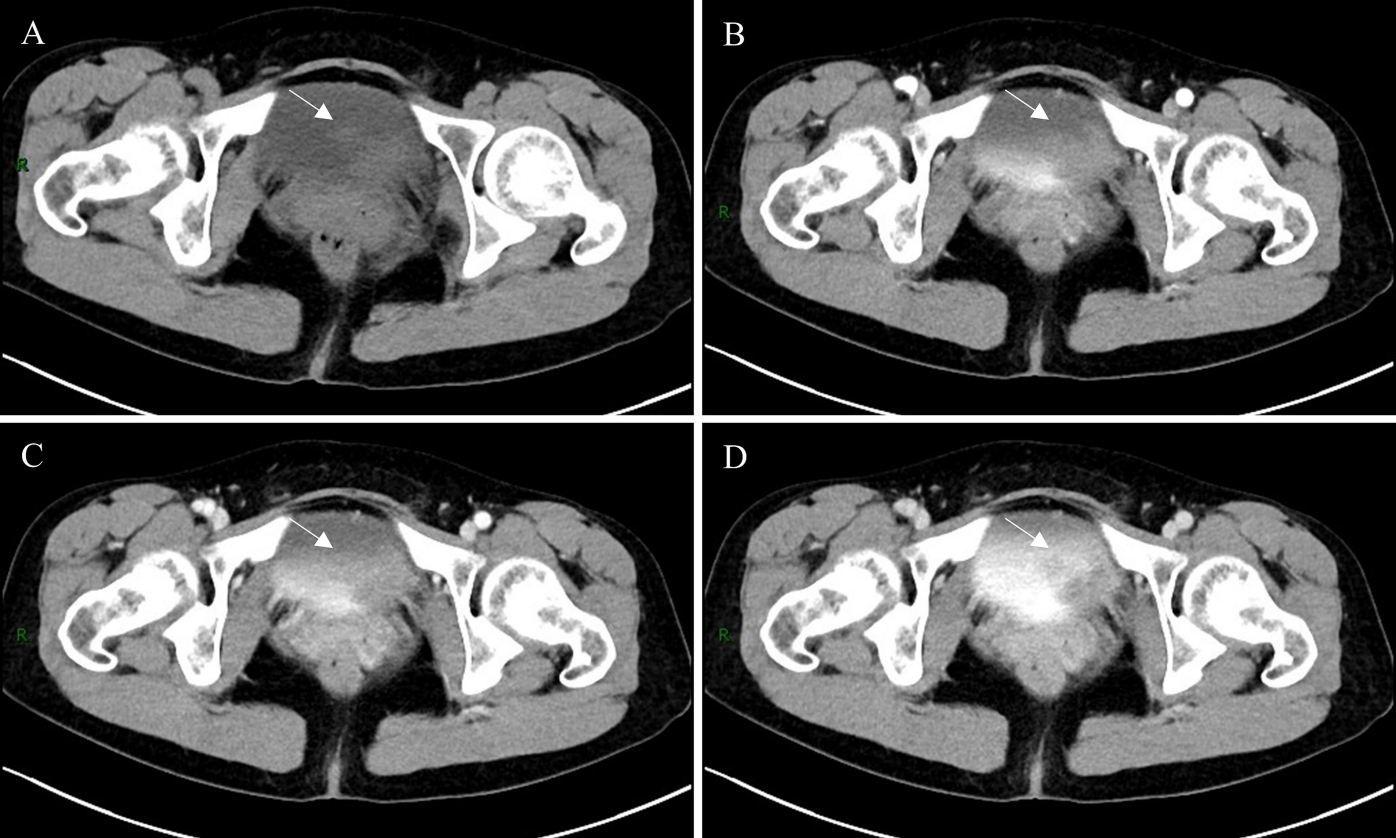
Non-contrast abdominal CT scan demonstrated an irregular soft tissue density lesion along the left lateral wall of the urinary bladder (**A**, arrow). The mass exhibited contrast enhancement during both arterial and venous phases (**B,C**, arrow), with subsequent excretory phase imaging revealing a focal filling defect in the corresponding region of the left lateral bladder wall (**D**, arrow).

**Figure 2:**
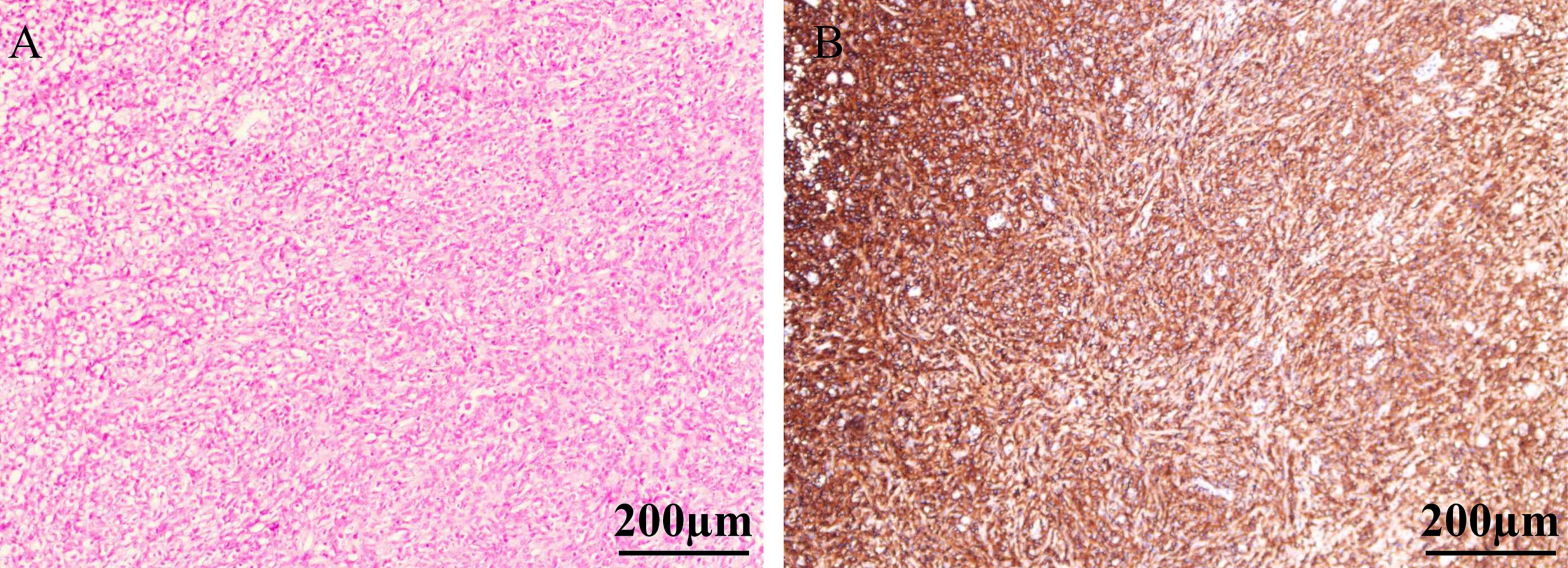
H&E staining revealed diffuse infiltration of the bladder lamina propria by a large number of atypical lymphoid cells, with near-complete replacement of the normal bladder tissue architecture **(A)**. The tumor cells exhibited diffuse strong positivity with brown staining on the cell membrane, indicating widespread expression of CD20 antigen **(B)**.

**Figure 3:**
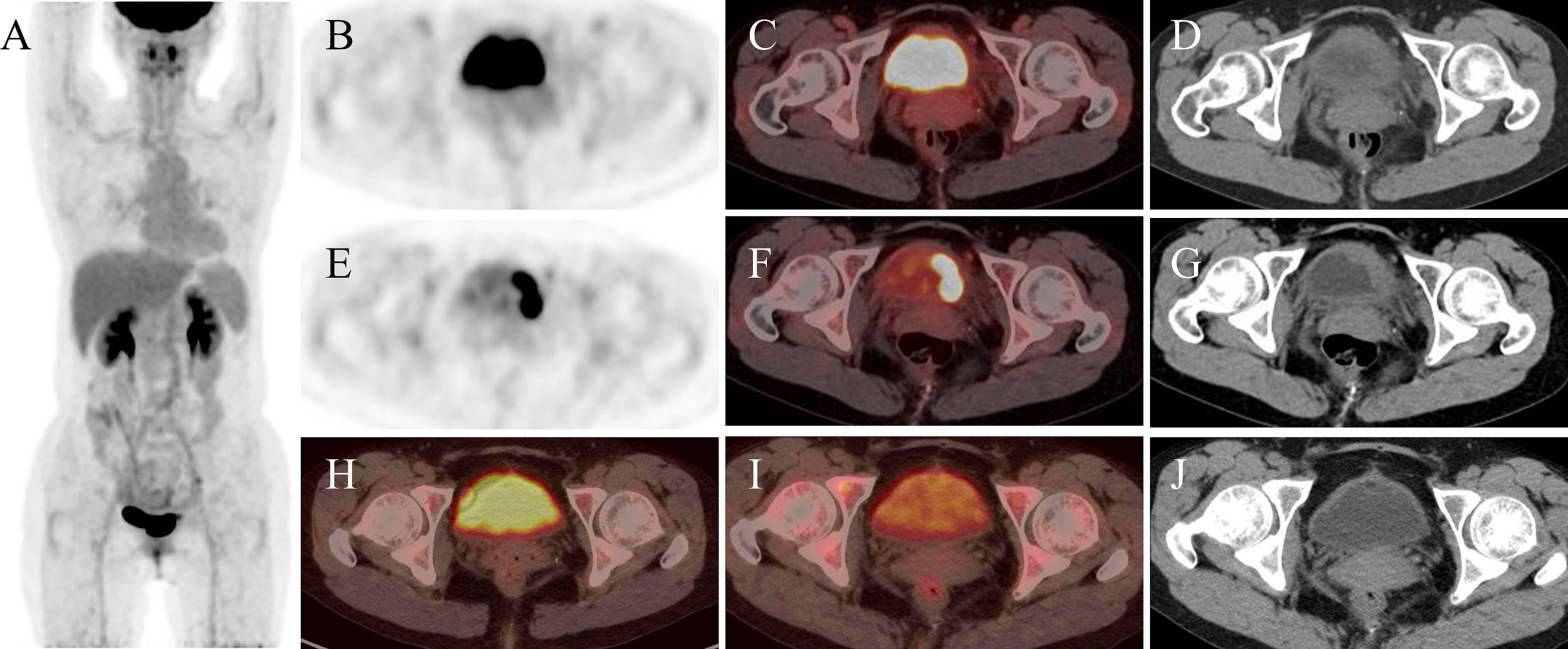
The whole-body ^18^F-FDG PET/CT. Maximum intensity projection (MIP) image prior to chemotherapy **(A)**. Axial PET, PET/CT fusion and CT before furosemide administration **(B–D)** demonstrate the lesion. Following intravenous furosemide administration, post-diuretic axial PET **(E)**, PET/CT fusion **(F)**, and CT **(G)** images reveal prominent delineation of the left lateral bladder wall lesion. Post-therapy evaluation after 4 cycles of R-CHOP regimen chemotherapy displays axial PET/CT fusion images of the bladder during standard acquisition **(H)** and subsequent delayed imaging at 1 h **(I)**, with comparison to pre-chemotherapy post-furosemide images demonstrating notably retained radiotracer activity within the bladder lumen. Corresponding post-chemotherapy CT image of the bladder is presented **(J)**.

A 50-year-old woman presented to the Urology Department in November 2024 with a 1-week history of gross hematuria and nocturia. She had no associated symptoms such as urinary frequency, dysuria, voiding difficulty, flank pain, or fever. The patient had a 6-year history of chronic hepatitis B, with no documented family history of lymphoma or hematologic malignancies. The patient’s vital signs were stable and superficial lymph nodes were not palpably enlarged. Urinalysis revealed nitrite positivity, leukocyturia (2 cells/μL), and occult blood (3 cells/μL). Serological testing for hepatitis B demonstrated: Hepatitis B surface antigen (HBsAg): Positive (>250,000 IU/mL), Anti-hepatitis Be antibody (Anti-HBe): Negative (0.02 S/CO), Anti-hepatitis B core antibody (Anti-HBc): Positive (7.88 S/CO), HBV DNA: Negative (<500 IU/mL). Routine urological color ultrasound examination suggested that a low and weak echogenic mass measuring about 33 mm × 20 mm was seen in the left wall of the bladder. Uro-pelvic CT scan and enhancement showed a mass on the left posterior wall of the bladder, which was considered to be a high possibility of bladder cancer (Figure 1). Cystoscopy revealed a mucosal bulge extending from the 12 to 5 o’clock position, involving the anterior bladder wall and left wall near the bladder neck, measuring approximately 40 mm × 30 mm. After exclusion of contraindications to surgery, “transurethral cystectomy” was performed. Postoperative pathology confirmed DLBCL of the bladder, Hans classification: anaplastic B-cell subset. Immunohistochemistry showed positivity for CD20, PAX-5, BcL-6, MUM1, Ki-67 (90%), and wild-type P53; negative for CD10, BCL-2, CD21, CD3, CD5, CK, c-Myc, CyclinD1, and GATA3 (Figure 2). To further evaluate disease staging, a PET/CT scan was performed after the transurethral cystectomy. Whole-body imaging performed 45 min after intravenous injection of ^18^F-FDG (185 MBq) showed diffusely increased bladder activity, which interfered with lesion detection. To reduce urinary radioactivity and enhance lesion conspicuity, pelvic delayed imaging was performed 60 min following intravenous furosemide injection (20 mg) at 120-min post-radiotracer administration. This revealed focal wall thickening with intense hypermetabolic activity with a SUVmax of 22.7 in the left posterior bladder wall (Figure 3), while no abnormal ^18^F-FDG uptake was detected in other organs or lymph nodes. Based on the above findings, the diagnosis was primary stage I DLBCL of the bladder. The patient was referred to another hospital in the city for further treatment. Bone marrow aspiration and biopsy revealed no evidence of lymphoma cells. After completing 4 cycles of R-CHOP (rituximab, cyclophosphamide, doxorubicin, vincristine, and prednisone) chemotherapy, a follow-up ^18^F-FDG PET/CT scan demonstrated no abnormal increased uptake in the bladder wall (Figure 3), indicating favorable treatment response. During follow-up, the patient remained asymptomatic with no hematuria and in good clinical condition, continuing to receive regular hospital treatments.

## Literature review

A comprehensive literature search was conducted in the Web of Science database to identify case reports and case series on primary bladder lymphoma published in English up to March 31, 2025. After systematic search and rigorous full-text review, secondary cases and publications lacking accessible full-text were excluded. The following data were extracted from eligible studies: first author, publication year, patient demographics (gender and age), clinical presentation, imaging findings, and follow-up outcomes (Table 1).

**Table 1:** Clinical and imaging features of the cases of primary bladder lymphoma from the literature review and current case.

Case no.	First author, year	Age (y)/sex	Symptoms	Location/bladder	US	CT/MRI	Cystoscopy	Bone marrow aspiration	Treatment	Recurrence
1 (13)	Jian-Hui Xin, 2024	83/F	Frequency urination	Posterior wall	Uneven hypoechoic lesion	CE: irregular enhancing nodule	Mild trigonal congestion, no neoplasia (post-treatment)	(−)	R-CHOP	(−)
2 (12)	Hideshige Seki, 2024	77/M	Fever, anorexia, general malaise, pyuria, bacteremia	NA	NA	Bladder wall thickening with perivesical stranding	NA	(−)	R-CHOP	(+)
3 (2)	Po-Sung Liang, 2024	79/F	Urinary frequency and urgency, suprapu-bic pain	Anterior wall	A suspicious 32 mm bladder tumor	CE: 39 mm polypoid enhancing lesion	Multiple bladder tumors	(−)	Radiation therapy	(−)
4 (11)	Xi Tu, 2023	81/F	Frequent urination and painful urination	Right side and posterior wall	A hypoechoic mass	Long T1 signal/short T2 signal, homogeneous CE enhancement, DWI restriction	Two nodular lesions	NA	TURBT	(−)
5 (10)	Atsuto Katano, 2022	65/F	Macroscopic hematuria, chronic cystitis	Bladder trigone	NA	Intermediate T2 signal intensity	A submucosal tumor	NA	TURBT, Radiation therapy	(−)
6 (9)	Zhen-Zhen Jiang, 2021	48/F	Macroscopic hematuria	NA	A large hypoechoic mass	Bladder wall thickening; ill-defined low-density nodules	Multiple bulging bladder masses	(−)	R-CHOP	(−)
7 (8)	Naoya Ishibashi, 2021	77/F	Hematuria and severe perineal pain	Bladder trigone	NA	Enlargement of an irregular elevated lesion	Non-papillary nodular tumor	NA	Radiation therapy	(−)
8 (5)	Rodolfo Montironi MD, 2015	81/M	Macroscopic hematuria, pain on micturition	Anterior wall to the right lateral bladder wall	NA	A solitary mass lesion	A wide-based mass	(−)	TURBT	(−)
9 (6)	Nischith D’Souza, 2017	56/M	Clot retention	Right lateral wall	A mass	NA	Polypoidal mass	NA	TURBT	(−)
10 (7)	Hewei Xue, 2020	77/F	Frequent urination, urinary urgency, dysuria	NA	Roughness in the inner walls of the bladder	Multiple nodules	Bladder occupying tumor	(−)	TURBT	(−)

US, ultrasound; CT, computed tomography; MRI, magnetic resonance imaging; CE, contrast-enhanced; NA, not applicable; F, female; M, male; T1, T1-weighted imaging; T2, T2-weighted imaging; DWI, diffusion-weighted imaging; TURBT, transurethral resection of bladder tumor; R-CHOP, rituximab + cyclophosphamide + hydroxydaunorubicin + oncovin + prednisone.

A total of 11 cases of primary bladder lymphoma, including the present case, were included in the analysis (2, 5–13). The cohort comprised 3 males (3/11) and 8 females (8/11), with a median age of 77 years (range 48–83). Mucosa-associated lymphoid tissue (MALT) lymphoma was the predominant histopathological subtype. Hematuria emerged as the most frequent clinical manifestation, followed by dysuria, urinary frequency, and recurrent urinary tract infections predominantly caused by *Escherichia coli*. Most lesions were localized to the lateral walls and trigone region of the bladder, presenting as single or multiple nodular lesions with occasional bladder wall thickening. Ultrasonographic evaluation revealed hypoechoic lesions demonstrating contrast enhancement and restricted diffusion on advanced imaging. Notably, none of the reviewed cases exhibited bone marrow infiltration. Treatment strategies encompassed chemotherapy, radiotherapy, and transurethral resection of bladder tumor (TURBT). In selected cases with localized disease, TURBT alone achieved therapeutic success. The majority of cases demonstrated favorable outcomes with standardized management, with only one case showing tumor recurrence during follow-up, which was associated with documented extravesical infiltration.

## Discussion

Approximately 40% of DLBCL cases originate from extranodal sites and the gastrointestinal tract representing the most common extranodal involvement (14, 15). While DLBCL demonstrates men predominance and higher incidence in individuals over 60 years, primary bladder lymphoma exhibits women preponderance (16). Notably, secondary bladder lymphoma occurs more frequently than its primary counterpart (1). The pathogenesis of DLBCL involves multifactorial mechanisms, with established risk factors encompassing genetic predisposition, immune dysregulation, and viral/environmental/occupational exposures (17). Recurrent urinary tract infections (UTIs) have been identified as a predisposing factor for primary bladder lymphoma (16, 18). Although our patient reported no definitive UTI symptoms or medical history, urinalysis revealed leukocyturia. Characteristic clinical manifestations of bladder lymphoma include gross hematuria, dysuria, urinary frequency, nocturia, and abdominal/back pain (16). The present case presented with gross hematuria and increased nocturnal voiding frequency. These nonspecific symptoms necessitate thorough differentiation from common urological conditions such as urinary tract infection, urothelial carcinoma, and mechanical obstruction.

The diagnosis of DLBCL of the bladder relies on imaging techniques, cystoscopy, and complete pathologic evaluation of tissue biopsies. On CT, bladder lymphoma may appear as an intraluminal nodular mass or focal wall thickening with early enhancement within approximately 60 s of contrast injection (19). In our patient, non-contrast CT revealed a nodular soft-tissue density in the left bladder wall with minimal enhancement on contrast imaging. During the excretory phase, a filling defect was observed against the high-density contrast background (Figure 1). Tumors on both T1 and T2-weighted images (T1WI and T2WI) on MRI show intermediate signal, and T1WI are suitable for detecting extravesical infiltration, lymph nodes, and bone metastases, while T2WI are suitable for assessing the depth of the tumor and differentiating the tumor from fibrosis, as well as for detecting peripheral organ invasion and bone marrow metastases (19). However, the limitations of CT and MRI imaging are difficult for the detection of metastatic lesions in normal-sized lymph nodes (20). ^18^F-FDG PET/CT demonstrates high sensitivity for lesion detection, particularly in identifying foci with no or minimal anatomical abnormalities on CT (21). This modality enables more accurate disease assessment and frequently alters lymphoma staging, consequently modifying therapeutic strategies (22). For patients with baseline PET-positive disease, serial monitoring of SUV changes and detection of new hypermetabolic lesions facilitate treatment response evaluation (22). Metabolic tumor volume (MTV), total lesion glycolysis (TLG), and maximal distance between lesions (Dmax) serve as critical prognostic parameters (23–25). Based on these advantages, we perform ^18^F-FDG PET/CT for suspected bladder lymphoma cases, which rapidly delineates extravesical involvement and determines whether the disease is secondary to nodal lymphoma. Some studies have shown that diuretic use significantly increases the lesion-to-bladder activity ratio, improving the detection rate (26–28). Moreover, dual-phase ^18^F-FDG PET/CT is of great clinical value for the staging of bladder cancer (29). Therefore, in the case of this patient, where the bladder lesion did not show well on early visualization (45 min after intravenous administration of ^18^F-FDG), a delayed visualization (2-h after intravenous administration of ^18^F-FDG) of 1 h after intravenous furosemide injection was taken (Figure 3). Before the use of diuretics, diffuse physiological ^18^F-FDG uptake was seen in the bladder due to physiological elimination of the radiotracer, which made it difficult to distinguish the metabolic distribution of the lesion on PET images. Following diuretic administration, PET imaging revealed a focal area of markedly increased metabolic activity in the left lateral wall of the bladder with a SUVmax of 22.7. This value significantly exceeded the average SUVmax of bladder cancer (16.1 ± 6.2) (30). Comparative analysis with the patient’s ^18^F-FDG PET/CT scan of post-chemotherapy (performed without intravenous diuretic bolus dual-phase imaging) demonstrated reduced background activity within the bladder after diuretic administration (Figure 3).

Although imaging studies provide preliminary differentiation between benign and malignant bladder lesions, histopathological examination remains essential for definitive diagnosis. MALT lymphoma/marginal zone lymphoma (MZL) has been historically regarded as the most common primary bladder lymphoma, while cases with extravesical extension are predominantly DLBCL (12, 31). DLBCL, not otherwise specified (NOS), represents the most frequent category (80–85% of DLBCL cases) and is histologically characterized by diffuse proliferation of large neoplastic cells effacing normal tissue architecture. These tumors demonstrate positivity for B-cell markers (CD20, CD79α, PAX5, CD22, CD19) and CD45 (1). Molecularly, DLBCL can be classified into two major subtypes by gene expression profiling: the germinal center B-cell-like (GCB) subtype and the activated B-cell-like (ABC) subtype (32). However, given the limited clinical availability of gene expression profiling, the immunohistochemistry-based Hans algorithm is routinely employed to categorize cases into GCB and non-GCB groups (the latter encompassing ABC subtypes and most unclassifiable cases) (14). In the present case, Hans classification indicated a non-germinal center B-cell phenotype. Histological examination revealed diffuse infiltration of the bladder lamina propria by sheets of atypical lymphoid cells with near-total effacement of normal bladder architecture. Immunohistochemistry confirmed CD20(+) and PAX-5(+) expression, establishing a B-cell lineage. A high Ki-67 proliferation index indicated aggressive tumor biology. These findings are consistent with DLBCL, leading to a definitive diagnosis of primary bladder diffuse large B-cell lymphoma.

DLBCL is a group of highly heterogeneous malignancies in terms of clinical manifestations, histomorphology and prognosis (1). The R-CHOP regimen remains the first-line treatment for DLBCL (1, 14, 33). However, its efficacy is suboptimal in elderly patients or those with comorbidities (34). For relapsed or refractory DLBCL, novel therapies such as chimeric antigen receptor T-cell (CAR-T) therapy and hematopoietic stem cell transplantation have emerged (34). Other approaches, including localized radiotherapy, BTK inhibitors, and immunomodulators, are primarily used as monotherapy or in combination to alleviate symptoms (35). The majority of primary bladder DLBCL cases (56.56%) were diagnosed at Stage I. Advanced age was identified as a poor prognostic factor, with patients aged ≥75 years exhibiting 2–3 times higher mortality than younger patients. The 5-year overall survival rate was 27.10% for patients ≥75 years versus 64.29% for those under 60 years (1). The patient has completed 4 cycles of R-CHOP chemotherapy. A follow-up ^18^F-FDG PET/CT scan revealed no increased uptake in the bladder wall lesion. Mildly increased uptake was observed in the bone marrow cavity due to post-chemotherapy bone marrow hyperplasia, with no other lesions showing elevated ^18^F-FDG uptake. Clinical evaluation indicated complete remission, and the patient is currently in good condition under regular treatment.

## Conclusion

Primary bladder DLBCL is a rare subtype of bladder lymphoma, predominantly affecting women. Dual-phase ^18^F-FDG PET/CT imaging significantly improves diagnostic accuracy for primary bladder lymphoma. When high bladder radiotracer activity causes imaging artifacts, a diuretic-enhanced washout protocol combined with controlled bladder distension can accelerate urine clearance and improve the target-to-background ratio.

## Data Availability

The original contributions presented in the study are included in the article/supplementary material, further inquiries can be directed to the corresponding author.
